# Knowledge and Expectations on Antibiotic Use among Older Adults in Malaysia: A Cross-Sectional Survey

**DOI:** 10.3390/geriatrics4040061

**Published:** 2019-10-25

**Authors:** Lai San Kong, Farida Islahudin, Leelavathi Muthupalaniappen, Wei Wen Chong

**Affiliations:** 1 Centre of Quality Management of Medicines, Faculty of Pharmacy, Universiti Kebangsaan Malaysia, Kuala Lumpur 50300, Malaysia; laisan_kong@hotmail.com (L.S.K.); faridaislahudin@ukm.edu.my (F.I.); 2 Department of Family Medicine, Medical Faculty, Universiti Kebangsaan Malaysia, Kuala Lumpur 50300, Malaysia; drleelaraj@yahoo.com

**Keywords:** older adults, antibiotic, knowledge, expectation

## Abstract

Antibiotics are commonly prescribed among older adults, and inappropriate use of antibiotics has been noted. However, there is limited information about their knowledge and expectations for antibiotics. This study aimed to assess older adults’ knowledge of antibiotic use and resistance, their expectations for antibiotics and the relationship between knowledge, expectation and inappropriate practices related to antibiotic use. A cross-sectional survey involving respondents aged 60 years and above was conducted, using a validated questionnaire. A lack of knowledge about the role of antibiotics was observed, whereby more than half of the respondents incorrectly believed that antibiotics can treat viral infections (53.5%) and colds and coughs (53.7%). Also, 67.9% of respondents incorrectly believed that antibiotic resistance occurs when the body becomes resistant to antibiotics. Almost half of the respondents would expect antibiotics for symptoms of self-limiting viral infections. Respondents who answered correctly for the role of antibiotics in viral infections were more likely not to expect antibiotics for cold, flu and cough (*p* < 0.001). Respondents who answered correctly regarding the need to adhere to antibiotics were more likely to have completed their antibiotic course (*p* < 0.001). Future educational initiatives should provide key information on the role of antibiotics and the importance of complying with antibiotics in this population.

## 1. Introduction

The devastating threat of antimicrobial resistance (AMR) to global human health is unquestionable [1]. Antimicrobials are losing their effectiveness in treating infections, due to the rapid emergence of AMR and the drying up of antimicrobial pipelines [2,3]. The difficult-to-treat AMR infections require longer hospital stays and more costly treatment, increase morbidity and mortality, and reduce productivity; therefore, increasing the economic burden of the countries [4]. In recent years, this global health crisis has been gaining much attention in Malaysia, and Malaysia has been striving hard to combat it via different approaches [5]. The major contributing factors of AMR are known to be overuse and misuse of antimicrobials [6].

Antibiotic consumption by the elderly population was found to be between 11% to 45%, with a significant rise over the past decade [7,8,9]. As much as 18% of the total antibiotic prescriptions in older adults was unnecessary, with 54% of them prescribed for acute respiratory conditions [8]. Healthcare providers face challenges in adequately diagnosing and managing infections in this vulnerable population because of the altered drug metabolism in this particular group, the common occurrence of drug–drug interactions due to polypharmacy and lower medication adherence [10,11]. Nursing homes or long term care facilities in the United States and Australia were found to be a reservoir for AMR pathogens due to excessive, inappropriate and prolonged broad-spectrum antimicrobial use, inadequate infection control and increased risk of pathogen colonization [12,13]. The prevalence of antimicrobial consumption in nursing homes was reported to be 11% in the United States, where only 66% of it was documented for therapeutic use [14].

The reported incidence of infections was higher in the older adult population because of the presence of chronic comorbidities, deteriorating organ functions and reduced host defence system [15,16]. Older adults also have poorer outcomes as a consequence of infections, including higher mortality, compared to adults of other age groups [17]. Atypical clinical presentations of infections, undertreatment or overtreatment, high risk of multidrug-resistant organism acquisition, rapid disease progression and prolonged recovery all contribute to poorer outcomes in the elderly [15,16]. Moreover, older adults who were living alone have lower health literacy and hence lower self-management abilities, contributing to poorer health [16,18,19].

A lack of knowledge on antibiotic use in the general public has been reported, with more than half of them who thought that antibiotics are useful in treating viral infections, and about one half of them would stop taking antibiotics when they felt better [20]. Those who were aware of antibiotic resistance were unaware that misuse of antibiotics could lead to this global crisis [20]. Those who had poor knowledge of antibiotic use were associated with a higher probability of antibiotic self-medication and practices of using leftover antibiotics, sharing antibiotics, keeping antibiotics for future use and stopping antibiotics when symptoms disappear [21,22]. Inappropriate practices related to antibiotics, including self-medication, were reported to be common among older adults [23]. In one study, a considerable proportion of older adults (77.8%) who had antibiotic use within the past six months were found to have experience of receiving antibiotics without prescriptions [21]. However, only a few studies reported the lack of knowledge and poor attitude on antibiotic use and resistance among older adults [24,25,26,27,28].

Patients who were unaware of the consequences of antibiotic overuse to the community were found to expect antibiotics from their prescriber [29]. Studies have found that those who incorrectly believed that antibiotics will work for viral infections were more likely to expect antibiotics from prescribers [30,31]. Patients’ expectations of antibiotics are found to be an important predictor of antibiotic prescribing [32,33]. Expectations for antibiotics for common cold were found among older adults when they felt sick enough to visit a physician, although most of them disagreed that antibiotics could speed the recovery [24]. 

To our knowledge, many studies have been done to assess the knowledge of antibiotic use and resistance; however, there has been limited research targeting vulnerable population groups, including older adults. Due to the paucity of information in this area, we conducted a study among the older adult population in Malaysia to assess their knowledge on antibiotic use and resistance and their expectations towards the need for antibiotics. This study also aimed to assess the relationship between knowledge, expectations and practices related to antibiotic use among older adults.

## 2. Materials and Methods

### 2.1. Study Design and Population

A cross-sectional study was conducted at a tertiary hospital in Kuala Lumpur from March 2019 to May 2019. Malaysian citizens aged 60 years and above were included in this study. Inpatients and those with cognitive impairment were excluded from the study. Respondents were recruited via convenience sampling. A minimum sample size of 382 was calculated using the below formula, based on a response distribution of 50%, confidence level of 95% and a margin of 5% error [34].

n = χ^2^NP(1 − P) / [d^2^(N − 1) + χ^2^P(1 − P)], (1)

where, n = required sample size; χ^2^ = the table value of chi-square for 1 degree of freedom at the desired confidence level (3.841); N = the population size (51593 based on 2018 data obtained from the hospital statistics); P = the population proportion (assumed to be 0.05 since this would provide the maximum sample size); d = the degree of accuracy expressed as a proportion (0.05).

### 2.2. Questionnaire Instrument

A questionnaire was developed based on literature review of other validated questionnaires from several studies [35,36,37,38]. The questionnaire comprised four sections. The first section contained demographic characteristics of the respondents. The second section was on respondents’ prior experience of antibiotic use, including recent antibiotic usage, source and reasons for taking antibiotics, which were assessed in accordance with the World Health Organization (WHO) multi-country public awareness survey [38]. This section also contained questions to assess inappropriate practices regarding antibiotic use, including self-medication, using leftover antibiotics, sharing antibiotics and early discontinuation of an antibiotic regimen. The third section consisted of a total of 20 items for which participants selected one answer option, which was “yes”, “no” or “not sure”. Among the 20 items, 12 assessed knowledge on antibiotic use and 8 assessed knowledge on antibiotic resistance. The questions on antibiotic use were taken from a previous study by Lim et al. [37] while the questions on antibiotic resistance were adopted from the WHO survey [38]. A knowledge score for each respondent was determined, whereby each correct answer was awarded one mark, and zero mark was given for each incorrect answer or answer with “not sure”, with a maximum total score of 20. The final section assessed respondents’ expectations towards antibiotic use and consisted of two parts, adapted with permission from Kuzujanakis et al. [36]. The first part was on respondents’ expectations to be prescribed with antibiotics for selected symptoms (7 statements) with response options of “yes”, “no” or “not sure”. The second part contained four statements which assessed respondents’ expectations from the physician in relation to antibiotic prescribing. The responses were assessed using a five-point Likert scale ranging from “strongly agree” to “strongly disagree”, which were then collapsed into three categories (agree, disagree and neutral).

The questionnaire was available in English and the national language (Bahasa Malaysia). It was first reviewed for content validity by an expert panel consisting of senior consultant specialists and pharmacists with expertise in infectious disease. A pilot study among 30 respondents was conducted to assess the clarity of the questions before finalizing the questionnaire. Internal consistency of the questionnaire using Cronbach’s α for the sections on knowledge and expectation were 0.686 and 0.829 respectively.

### 2.3. Data Collection

Adults who were available in the hospital area were screened via inclusion and exclusion criteria. Potential elderly respondents were identified and approached by the researchers, and the purpose of the study was explained before their participation. After obtaining written consent, the questionnaire was given to the participants for completion. Assistance was provided for those who requested for it by the researcher. No personal identifiers were included in the form. Respondent participation was voluntary, and their responses were dealt with a high level of confidentiality and anonymity. Ethical approval was obtained from the hospital ethics committee (reference number: UKM PPI/111/8/JEP-2018-717), before proceeding to the data collection phase.

### 2.4. Data Analysis

Data analyses were performed using IBM SPSS version 18. Descriptive statistics were used to summarize the data. The independent *t*-test was used to address the difference in total knowledge scores regarding respondent expectation for antibiotics. The chi-squared test was used to assess the association between respondents’ knowledge on antibiotic effect with their expectation to be prescribed with an antibiotic for colds, flu and cough.

In addition, independent *t*-test was also used to assess the difference in total knowledge scores between those who reported having had at least one inappropriate practice and those with no inappropriate practice. The chi-squared test was used to assess the association between two subitems of the knowledge section (“It is okay to stop taking antibiotics when symptoms are improving” and “Taking less antibiotics than prescribed is healthier than taking the full course prescribed”) with the practice of completing the antibiotic course. Chi-squared tests were also used to assess the association between demographic characteristics (age, gender, ethnicity, educational level and prior receipt of antibiotics in the past year) and knowledge of antibiotics. For this analysis, adequate and inadequate knowledge were defined as total knowledge scores of >10 and <10, respectively. A *p-*value of <0.05 with confidence interval of 95% was considered statistically significant for all tests.

## 3. Results

A total of 402 participants were recruited. Demographic characteristics of respondents are shown in Table 1. There was an equal distribution of male and female respondents, with a mean age of 67.72 ± 5.77 years old. The majority of the respondents were Malay (58.0%), had completed secondary education (86.5%), were retired (65.4%) and currently have a previous occupation not related to healthcare (97.8%).

### 3.1. Prior Experience and Practices in Antibiotic Use

Respondents’ prior experiences and practices related to antibiotic use are presented in Table 2. A total of 13.9% of the respondents reported receiving antibiotics in the past month, and 18.7% of them received antibiotics in the last six months. Of those who took antibiotics, almost all of them (96.9%) were prescribed by a physician. The main reason for taking antibiotics was for a respiratory tract infection (35.4%), followed by fever (19.4%) and pain or inflammation (14.2%). A small proportion of them could not remember (4.2%) or did not know (2.1%) the indication of the antibiotics. Most of them (95.7%) received advice on how to take antibiotics from healthcare professionals, mainly physicians (74.6%) and followed by pharmacists (15.0%).

Low rates of inappropriate practices in antibiotic use were observed, whereby only 3.5% of respondents had ever obtained antibiotics without a prescription, 3.5% used own/others’ leftover antibiotics without advice from healthcare professionals and 2.5% shared own leftover antibiotics with friends or family or neighbours. The most common inappropriate practice was not completing antibiotics according to the duration prescribed, which was reported by 17.2% of the respondents (Table 2).

### 3.2. Knowledge about Antibiotic Use and Resistance

The respondents scored an average of 6.6 (± 2.4) out of a total score of 12 in knowledge on antibiotic use and 3.6 (± 1.8) out of a total score of 8 in knowledge on antibiotic resistance, with the total score on both sections 10.2 (± 3.5) out of a total score of 20. A high proportion of respondents (70.4%) answered correctly that antibiotics are medicines that can be used to kill bacteria (Table 3). However, more than half of the respondents also incorrectly believed that antibiotics can be used to treat viral infections (53.5%) and that antibiotics work on most colds and coughs (53.7%). Moreover, 48.3% of them thought that antibiotics are the same as medications used to relieve pain and fever, such as aspirin and paracetamol, and only about one-third (32.1%) correctly identified penicillin as an antibiotic. Nevertheless, a high proportion of them were aware that they should continue taking antibiotics even when their symptoms are improving (79.6%) and that overuse of antibiotics can lead to its reduced effectiveness (64.9%) (Table 3).

In terms of knowledge on antibiotic resistance, 67.9% of respondents incorrectly believed that antibiotic resistance occurs when the body becomes resistant to antibiotics, and 62.4% of them thought that antibiotic resistance is only a problem for people who take antibiotics regularly. About half of the respondents either did not believe (30.8%) or were not sure (20.6%) that antibiotic resistance could affect them or their family (Table 3).

### 3.3. Expectations for Antibiotic Treatment

**Figure 1:**
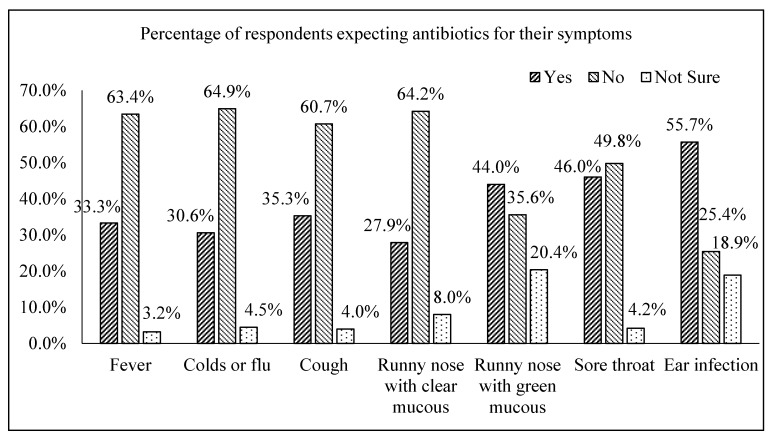
Percentage of respondents who expected antibiotics to be prescribed for the listed common symptoms (n = 402).

The respondents’ expectations to be prescribed with antibiotics for common symptoms were shown in Figure 1. Almost half of the respondents would expect antibiotics to be prescribed for runny nose with green mucus (44.0%), sore throat (46.0%) and ear infection (55.7%). Approximately one-third of them would expect an antibiotic for their fever (33.3%), cold or flu (30.6%), cough (35.3%) and runny nose with clear mucous (27.9%).

Only a small proportion of respondents agreed or strongly agreed that they would be less satisfied with the physician visit if they do not receive an antibiotic (12.44%) or that they will visit another physician if the physician does not prescribe an antibiotic (18.66%). A total of 83.4% agreed or strongly agreed that they expect the physician to discuss with them regarding the need for an antibiotic for their symptoms, rather than just giving them a prescription (Table 4).

### 3.4. Association between Knowledge, Expectations and Practice

Overall, regarding respondents’ expectations towards antibiotic prescribing by their physicians, knowledge of antibiotics showed significant effect for only one subitem. The respondents who strongly agreed or agreed that they would be less satisfied with the physician visit if they do not receive an antibiotic were found to have significantly higher mean knowledge scores (11.2 ± 3.245) compared to those with other responses (10.05 ± 3.522), with *p* = 0.030 (Table 4). However, respondents who answered correctly for the statements on antibiotic effect (“Antibiotics can be used to treat viral infections” and “Antibiotics work on most colds and coughs”) were more likely not to expect antibiotics for colds, flu or cough (*p* < 0.001) (Table 5).

When comparing knowledge scores between respondents with at least one inappropriate practice and those with no inappropriate practice, the difference was not significant (*p* = 0.577). However, respondents who answered correctly for the statements (“It is okay to stop taking antibiotics when symptoms are improving” and “Taking less antibiotics than prescribed is healthier than taking the full course prescribed”) were more likely to have completed their antibiotic course (*p* < 0.001) (Table 6).

### 3.5. Factors Associated with Knowledge of Antibiotics

As shown in Table 7, age was significantly associated with knowledge of antibiotics, whereby respondents who are older tended to have inadequate knowledge (*p* = 0.007). In addition, there was a significant association between educational level and knowledge, whereby respondents with higher educational level tended to have adequate knowledge (*p* < 0.001). There were no significant associations between gender, ethnicity and prior receipt of antibiotics with knowledge.

## 4. Discussion

The findings in this study point to a lack of knowledge about the role of antibiotics and awareness about the impact of antibiotic resistance among the older adult population in Malaysia. Moreover, this study revealed inappropriate expectations for antibiotic prescriptions for symptoms related to self-limiting viral infections in some of the respondents.

A substantial proportion of older adults in this study reported using antibiotics within the past 12 months (42.8%). However, the percentage of respondents using antibiotics within the past month (13.9%) was lower compared to previous studies conducted among the general public in Malaysia and among the elderly in a multi-country survey by WHO [35,37,38,39,40]. Nevertheless, the main reasons cited for taking antibiotics in this study, which were respiratory tract infections (35.4%) and fever (19.4%), were similar to previous surveys in Malaysia [35,40,41] and in other countries [42,43]. Similar to a previous study among the general public in Penang [35], the majority of respondents (96.9%) were prescribed the antibiotic after consultation with the physician. Only a small proportion (1.5%) of the respondents received antibiotics without physician consultation. This self-medication rate was comparatively much lower than those reported in other studies conducted among adults, which were 5.3% to 20.4% in Malaysia [35,39,40,41,44], and 22.4% to 76.6% in other countries [42,43,45,46,47,48,49], in which respondents obtained antibiotics from the pharmacy without prescriptions, followed by antibiotics shared by family or friends, and purchased from clinics without consultation. A systematic review reported that age is not a strong determinant in self-medication with antibiotics [50]; therefore, the lower rate of self-medication in this study might not be due to subject selection for elderly population. A potential reason for the low rate of self-medication in this study is the recruitment of respondents from a public tertiary hospital, where medications are supplied with prescriptions. It could be possible that most of the respondents usually received their health care from public hospitals or health clinics.

Inappropriate practices related to antibiotic use were relatively low compared to other studies. Only 3.5% of the respondents in this study had obtained antibiotics without prescription, which was comparable to another Malaysian study in Putrajaya (4.5%) [37], but lower than that reported in Hong Kong (9%) [51] and in Lithuania (27.8%) [47]. The percentage of respondents who had used leftover antibiotics is 3.5%, and this rate was similar to a study in Korea [52], but lower that that reported in India (34%) [49]. Similarly, a low rate of sharing own leftover antibiotics to friends or family or neighbors (2.5%) was found in this study, as compared to 26.5% in Lebanon [45], 31% in India [49] and 40% in Omani [53]. The most common inappropriate practice reported in this study was not completing antibiotic course according to the duration prescribed (17.2%), but this rate was also lower than that reported in adults in other countries (32–50%) [24,26,42,43,45,48,54,55]. Despite the lower rates reported in this study compared to the other studies conducted in the general population, inappropriate practices in older adults still warrant attention. In addition to contributing to the development of antibiotic resistance, there is a higher risk of acquisition of multidrug-resistant organisms and increased risk of mortality from AMR in this vulnerable population, as a result of impaired immune system and more frequent healthcare system contact due to chronic morbidities [10].

In terms of knowledge regarding antibiotic use, respondents in this study were found to be confused with the role and identification of antibiotics, a finding that is in line with other Malaysian studies [28,35,37,39,40,41,56]. Although 70.4% of respondents in this study answered correctly that antibiotics are medicines that can kill bacteria, only a small proportion of respondents (18.9%) were able to correctly identify antibiotics as being ineffective against viral infections. This finding is comparable to previous studies in Malaysia, whereby more than 80% of adult respondents in Putrajaya [37] and Penang [35], and elderly respondents in Cheras [28] also failed to identify that antibiotics do not eradicate viral infections. The finding of the misconception that antibiotics work on both bacterial and viral infections was not uncommon in other studies in the world [26,30,31,38,43,45,48,57,58], because the general public incorrectly believed that antibiotics can speed up the recovery from most cough and cold [21,24,30,45,48,52]. In addition, more than half of the respondents in this study were not able to differentiate antibiotics from other commonly used medications such as analgesics and antipyretics, similar to that reported in Penang (69.1%) [35]. A possible reason for these gaps in knowledge was the lack of explanation provided to patients regarding antibiotics and the difference between bacterial and viral infections. Previous work has discussed the benefits of using the specific terms “bacteria” and “virus” when providing medical consultations to patients [57]. In addition, frequent prescribing of antibiotics in viral infections and inconsistency in antibiotic prescribing by different prescribers for the same symptoms might have caused confusion to older adults [31].

Respondents were aware of the problem of antibiotic resistance and were able to make connection between antibiotic use and resistance. However, misinterpretations were noted, whereby a majority of them (67.9%) as compared to 76% of the general public in WHO multi-country survey [38], viewed that antibiotic resistance occurred when “the body gets used to it”, in relation to the body’s immune system for the antibiotic to work effectively instead of as a property of infecting organisms [59]. In addition, responses also indicated a low perceived threat to antibiotic resistance, whereby less than half of the respondents (48.5%) believed that antibiotic resistance is an issue that could affect them or their family. About 62.4% of respondents in this study incorrectly believed that antibiotic resistance is only a problem for people who take antibiotics regularly, compared to only 49.6% of the elderly respondents in Hong Kong [60]. This is of concern, as perceived low threat of antibiotic resistance may result in low sense of ownership and individual responsibility in contribution to the cause and control of bacterial resistance [61]. Despite the efforts to raise awareness of antibiotic resistance among the public, the misconceptualizations and negative perceived individual contributions found in this study implied that there may be a difference in the public’s interpretation and understanding on the information received [59,61]. Therefore, there is a need to design effective communication messages or framework to better deliver the concepts of antibiotic resistance in public health campaigns, so as to improve the public’s understanding and engage them in the combat of antibiotic resistance [59,61].

Commonly, antibiotics were incorrectly believed by the public to be useful for symptoms in viral infections such as fever, cough, sore throat, colds or flu [30,58]. This is similar to our study, whereby about a third of the respondents would expect an antibiotic prescription for the treatment of their fever, colds or flu, and cough. These inaccurate expectations are associated to their lack of knowledge towards the correct indication of antibiotics. Patients’ expectations towards the need for antibiotics are important, as studies have shown that physicians can be pressured by patients for unnecessary antibiotic prescriptions [30,62]. However, as compared to this study, the proportions of Malaysian adult respondents in previous studies who expected antibiotics to be prescribed for their cold were higher (47.3% in Penang [35], 57.4% in Shah Alam [40] and 73.8% in Putrajaya [37]). This finding was in line with previous studies that found older adults to be less likely to expect or demand an antibiotic for their colds or flu [27,28,30,42].

In addition, responses in this study also suggested that majority of older adults trusted the physicians’ decisions regardless if antibiotics were prescribed to them, which was in line to that reported in a local study [28]. Elderly respondents were found to have a higher level of trust and confidence in antibiotic prescribing decisions by their physicians compared to younger respondents [62]. However, a majority of the respondents in this study (83.4%) also expected the physician to discuss with them regarding the need of antibiotics for their symptoms, rather than just giving them a prescription. This indicates that older adults wanted their prescribers to explain to them about the need of antibiotics for their illness, and to share decision making in an easily understandable way [31,60]. Contrary to this, the elderly respondents in Hong Kong reported that they did not want physicians to discuss with them regarding the need of antibiotic prescriptions [60]. Healthcare providers were also regarded as the main sources of information pertaining to antibiotics among the respondents in this study, highlighting their important role in influencing antibiotic knowledge and practices for this group of population.

This study did not find a significant difference in total knowledge scores between respondents with at least one inappropriate practice and those with no inappropriate practice. In their study, McNulty et al. also concluded that there was no simple relationship between knowledge, attitude and practice on the prudent use of antibiotics [25]. However, in this study, respondents with better knowledge regarding the need to adhere to antibiotic regimen were more likely to have completed their antibiotic course, which is in line with another Malaysian study [41]. In addition, significant associations were obtained between respondents’ knowledge on antibiotic effect and their expectations of antibiotic treatment for the common cold or cough. This finding corroborated well with the results of other studies, which also found that respondents who incorrectly believed that viral infections or cold and cough can be treated with antibiotics, had a higher probability to expect an antibiotic prescription for their cough and cold [30,31]. The findings in the present study support the idea that provision of key information on the indication of antibiotics and importance of complying with antibiotic regimen, can influence respondents’ expectations and their proper use of antibiotics. Therefore, it is imperative to emphasize these important key messages in any educational campaigns.

In this study, age and educational level were found to have significant associations with respondents’ knowledge on antibiotic use and resistance. Respondents who were older and with fewer educational qualifications were less knowledgeable about antibiotics. These findings were in line with some studies conducted in Malaysia and other countries [26,37]. In a study by McNulty et al. [25], a higher general level of education is strongly associated with a better knowledge about the effectiveness of antibiotics. However, poor understanding and learning abilities are common in older adults; therefore, specific models and considerations should be taken into account in designing educational materials and campaigns for this population in order to ensure effective delivery of key messages [63]. Educational interventions via older adult-specific, evidence-based interactive health literacy programs have been reported to improve decision self-efficacy and health literacy skills among older adults [64,65].

The strength of this study was that it was conducted in an under-represented population in this research area. However, there were a few limitations in this study. First, this study was conducted in a single centre, and therefore the results may not be generalizable to the whole elderly population in Malaysia. Future research can be conducted in community or nursing home settings to enable comparison of findings among different settings, so as to aid in the local tailoring of planned actions. Secondly, the use of convenience sampling method may have resulted in the possibility of selection bias during the recruitment of respondents. Furthermore, as with all self-reported data, there was also the potential for recall and response bias.

## 5. Conclusions

Although low rates of inappropriate antibiotic use practices were reported in this study, this study identified several gaps in important areas of knowledge on antibiotic use, particularly on the role and identification of antibiotics. In addition, incorrect beliefs about antibiotic resistance were observed among older adults. Provision of key information on indication of antibiotics may influence respondents’ expectations toward antibiotics, and information on the importance of complying with antibiotic regime may influence their correct use of antibiotics. These findings should be considered when designing suitable educational interventions or local campaigns which are older adult-specific, to correct the misunderstandings in antibiotic use and resistance for this group of population.

## Figures and Tables

**Table 1:** Demographic characteristics of respondents (n = 402).

Respondents’ Characteristics	n (%)
Age (mean ± SD)	67.72 ± 5.77
Gender	
Male	201 (50.0%)
Female	201 (50.0%)
Ethnicity	
Malay	233 (58.0%)
Chinese	111 (27.6%)
Indian	55 (13.7%)
Others	3 (0.7%)
Educational level	
No formal education	4 (1.0%)
Primary education	50 (12.4%)
Secondary education	228 (56.7%)
Pre-university education	44 (10.9%)
Tertiary education	76 (18.9%)
Main occupation	
Unemployed	19 (4.7%)
Employed for wages	10 (2.5%)
Self-employed	23 (5.7%)
Housewife/Househusband	87 (21.6%)
Retiree	263 (65.4%)
Occupation related to healthcare	
Yes	9 (2.2%)
No	393 (97.8%)
Monthly income	
None	224 (55.7%)
<RM 1000	21 (5.2%)
RM 1000–RM 3999	115 (28.6%)
RM 4000–RM 6999	31 (7.7%)
RM 7000–RM 10,000	3 (0.7%)
≥RM 10,000	8 (2.0%)

Abbreviation: SD = standard deviation.

**Table 2:** Prior experience in antibiotic use.

	n (%)
**Last take antibiotics** (n = 402)	
In the last month	56 (13.9%)
In the last 6 months	75 (18.7%)
In the last year	41 (10.2%)
More than a year ago	85 (21.1%)
Never	12 (3.0%)
Cannot remember	133 (33.1%)
**Source of antibiotics** (n = 257)	
Prescribed after consultation with physician	249 (96.9%)
Prescribed after consultation with dentist	4 (1.6%)
Retail pharmacy/community pharmacy	3 (1.2%)
Leftover antibiotics (own/someone’s else)	1 (0.3%)
**Reason(s) to take antibiotics** (n = 257)	
Fever	56 (19.4%)
Pain/inflammation	41 (14.2%)
Respiratory tract infection	102 (35.4%)
Urinary tract infection	16 (5.6%)
Skin problems/wounds/cuts	21 (7.3%)
Dental problem	4 (1.4%)
Diarrhoea/vomiting	3 (1.0%)
Others	27 (9.4%)
Do not know	6 (2.1%)
Cannot remember	12 (4.2%)
**Get advice on how to take antibiotics** (n = 257)	
Yes	246 (95.7%)
No	11 (4.3%)
**Source(s) of advice** (n = 257)	
Physician	229 (74.6%)
Dentist	3 (0.9%)
Pharmacist	46 (15.0%)
Nurse	7 (2.3%)
Friends/family/neighbours	15 (4.9%)
Patient information leaflet	5 (1.6%)
Mass media	2 (0.7%)
**Inappropriate practice**	
Obtained antibiotics without a prescription (n = 402)	
Yes	14 (3.5%)
No	388 (96.5%)
Used own/others’ leftover antibiotics without advice from healthcare profession (n = 402)	
Yes	14 (3.5%)
No	388 (96.5%)
Not completed antibiotic course (n = 402)	
Yes	69 (17.2%)
No	333 (82.8%)
Shared own leftover antibiotics to friends or family or neighbours (n = 402)	
Yes	10 (2.5%)
No	392 (97.5%)
Engaged in at least one inappropriate practice (n = 402)	
Yes	83 (20.65%)
No	319 (79.35%)

**Table 3:** Knowledge about antibiotic use and resistance (n = 402).

			Respondents’ Answer, n (%)		
			Correct	Incorrect	Not Sure
	**Knowledge about antibiotic use**				
1.	Antibiotics are medicines that can kill bacteria.	True	283 (70.4%)	25 (6.2%)	94 (23.4%)
2.	Antibiotics can be used to treat viral infections.	False	76 (18.9%)	215 (53.5%)	111 (27.6%)
3.	Antibiotics work on most colds and coughs.	False	146 (36.3%)	216 (53.7%)	40 (10.0%)
4.	Antibiotics can kill bacteria that normally live on the skin and gut (digestion tract).	True	220 (54.7%)	60 (14.9%)	122 (30.3%)
5.	Bacteria that normally live on the skin and in the gut are good for your health.	True	229 (56.9%)	97 (24.1%)	76 (18.9%)
6.	Antibiotics are the same as medications used to relieve pain and fever such as aspirin and paracetamol (Panadol).	False	179 (44.5%)	194 (48.3%)	29 (7.2%)
7.	Penicillin is an antibiotic.	True	129 (32.1%)	122 (30.3)	151 (37.6%)
8.	Antibiotics may cause allergy reactions.	True	265 (65.9%)	79 (19.7%)	58 (14.4%)
9.	All antibiotics do not cause side effects.	False	223 (55.5%)	114 (28.3%)	65 (16.2%)
10.	It is okay to stop taking antibiotic when symptoms are improving.	False	320 (79.6%)	72 (17.9%)	10 (2.5%)
11.	Taking less antibiotics than prescribed is healthier than taking the full course prescribed.	False	333 (82.8%)	54 (13.4%)	15 (3.7%)
12.	Overuse of antibiotics can cause the antibiotics to lose effectiveness in long term.	True	261 (64.9%)	71 (17.7%)	70 (17.4%)
	**Knowledge about antibiotic resistance**				
1.	Antibiotic resistance occurs when your body becomes resistant to antibiotics and they no longer work as well.	False	60 (14.9%)	273 (67.9%)	69 (17.2%)
2.	Many infections are becoming increasingly resistant to treatment by antibiotics.	True	246 (61.2%)	68 (16.9%)	88 (21.9%)
3.	If bacteria are resistant to antibiotics, it can be very difficult or impossible to treat the infections they cause.	True	288 (71.6%)	43 (10.7%)	71 (17.7%)
4.	Antibiotic resistance is an issue that could affect me or my family.	True	195 (48.5%)	124 (30.8%)	83 (20.6%)
5.	Antibiotic resistance is an issue in other countries but not here.	False	240 (59.7%)	40 (10.0%)	122 (30.3%)
6.	Antibiotic resistance is only a problem for people who take antibiotics regularly.	False	72 (17.9%)	251 (62.4%)	79 (19.7%)
7.	Bacteria which are resistant to antibiotics can be spread from person to person.	True	167 (41.5%)	143 (35.6%)	92 (22.9%)
8.	Antibiotic-resistant infections could make medical procedures like surgery, organ transplants and cancer treatment much more dangerous.	True	180 (44.8%)	56 (13.9%)	166 (41.3%)

**Table 4:** Respondents’ mean knowledge score and expectation on antibiotic use (n = 402).

		n (%)	Mean Knowledge Score (±SD)	*p*-value
If I expect an antibiotic, I am less satisfied with the physician visit if I do not receive one.	Agree	50 (12.44%)	11.2 ± 3.245	0.030 *
	Disagree/ neutral	352 (87.56%)	10.05 ± 3.522	
I would rather take an antibiotic that may not be needed than wait to see if I will get better without it.	Agree	48 (11.94%)	9.38 ± 3.751	0.086
	Disagree/ neutral	354 (88.06%)	10.30 ± 3.462	
If a physician does not prescribe an antibiotic when I think one is needed, I will visit another physician.	Agree	75 (18.66%)	10.95 ± 3.238	0.0928
	Disagree/ neutral	327 (81.34%)	10.02 ± 3.546	
I expect the physician to discuss with me regarding the need of an antibiotic for my symptoms, rather than just giving me a prescription.	Agree	335(83.40%)	8.95 ± 4.307	0.053
	Disagree/ neutral	67 (16.60%)	10.33 ± 3.381	

Abbreviation: SD = standard deviation, * = significant *p* value.

**Table 5:** Respondents’ knowledge versus their expectations to be prescribed antibiotic for their cold, flu or cough (n = 402).

		Expectations to be Prescribed Antibiotic for Their Cold, Flu or Cough, n (%)		χ^2^, *p*-value
		Yes	No	
Antibiotics can be used to treat viral infections.	Answered correctly	6 (8.1%)	68 (91.9%)	χ^2^ = 15.576, *p* < 0.001*
	Answered incorrectly/ not sure	100 (30.5%)	228 (69.5%)	
Antibiotics work on most colds and coughs.	Answered correctly	8 (5.4%)	139 (94.6%)	χ^2^ = 52.267, *p* < 0.001*
	Answered incorrectly/ not sure	98 (38.4%)	157 (61.6%)	

* = significant *p* value.

**Table 6:** Respondents’ knowledge versus their inappropriate practice of not completing antibiotic prescribed (n = 402).

		Inappropriate Practice of Not Completing Antibiotics Prescribed, n (%)		χ^2^, *p*-value
		Yes	No	
It is okay to stop taking antibiotics when symptoms are improving.	Answered correctly	26 (8.1%)	294 (91.9%)	χ^2^ = 90.153, *p* < 0.001*
	Answered incorrectly/ not sure	43 (52.4%)	39 (47.6%)	
Taking less antibiotics than prescribed is healthier than taking the full course prescribed.	Answered correctly	40 (12.0%)	293 (88.0%)	χ^2^ = 36.221, *p* < 0.001*
	Answered incorrectly/ not sure	29 (42.0%)	40 (58.0%)	

* = significant *p*-value.

**Table 7:** Association between factors and respondents’ knowledge on antibiotics (n = 402).

Factors	n (%)		χ^2^, *p*-value
	Adequate Knowledge (≥10)	Inadequate Knowledge (<10)	
Age			
60–69 years old	163 (61.7%)	101 (38.3%)	χ^2^ = 9.979, *p* = 0.007*
70–79 years old	68 (58.6%)	48 (41.4%)	
≥80	6 (27.3%)	16 (72.7%)	
Gender			
Male	124 (61.7%)	77 (38.3%)	χ^2^ = 1.244, *p* =0.265
Female	113 (56.2%)	88 (43.8%)	
Ethnicity			
Malay	140 (60.1%)	93 (39.9%)	χ^2^ = 2.289, *p* = 0.318
Chinese	68 (61.3%)	43 (38.7%)	
Indian and others	29 (50.0%)	29 (50.0%)	
Educational level			
No formal education and primary education	23 (42.6%)	31 (57.4%)	χ^2^ = 16.07, *p* < 0.001*
Secondary education	127 (55.7%)	101 (44.3%)	
Pre-university and tertiary education	87 (72.5%)	33 (27.5%)	
Last receipt of antibiotics within the past 6 months			
Yes	73 (55.7%)	58 (44.3%)	χ^2^ = 0.838, *p* = 0.360
No	164 (60.5%)	107 (39.5%)	
Last receipt of antibiotics within the past 1 year			
Yes	103 (59.9%)	69 (40.1%)	χ^2^ = 0.107, *p* = 0.743
No	134 (58.3%)	96 (41.7%)	

* = significant *p* value.
